# Right ventricular 2D speckle-tracking echocardiography in children with osteosarcoma under chemotherapy

**DOI:** 10.1186/s43044-019-0028-9

**Published:** 2019-11-21

**Authors:** Ibtsam Khairat, Mohamed Khalfallah, Aliaa Shaban, Ibrahim Abu Farag, Asmaa Elkady

**Affiliations:** 1 Cardiology Department, Faculty of Medicine, Tanta University, Tanta, Egypt; 2 Pediatric Deparment, Faculty of Medicine, Al_Azhar university, Cairo, Egypt; 3 Oncology Department, Faculty of Medicine, Tanta University, Tanta, Egypt

**Keywords:** Right ventricle, speckle-tracking echocardiography, Osteosarcoma, Chemotherapy

## Abstract

**Background:**

Cardiotoxicity from anthracyclin chemotherapy is a leading cause of death in patients with cancer. Therefore, left ventricular (LV) function is routinely assessed during protocol to detect cardiotoxicity; however, animal studies suggest that right ventricular (RV) function may be also impaired. So, our objective was to investigate the incidence of RV dysfunction in children with osteosarcoma receiving anthracyclines and to highlight the role of 2D STE in early detection of RV dysfunction.

**Results:**

RV function was affected by anthacyclines through direct cardiotoxic effect on RV myocardium without simultaneous derangement of LV function. Furthermore, there is a direct proportion between the incidence of RV dysfunction and the cumulative dose of anthracyclines. At the first echo follow-up at 10th week, 7 patients had impaired RV GLS in comparison to baseline study. At 20th week, the number of patients with impaired RV strain increased to 10. At 29th week, it reaches 12 patients. This effect was early detected by RV 2DSTE before adversely affecting TAPSE and FAC. The incidence of RV dysfunction from anthracyclines was around 12%, and the recovery rate was around 8% in 3 months after completion of chemotherapy.

**Conclusion:**

RV 2DSTE is the best modality to detect early affection of RV function in comparison with other modalities. RV function decreases early even before derangement of LV function. Accordingly, it should be assessed separately in all patients who received anthracyclines even without evident LV affection.

## Background

Osteosarcoma is considered the most common primary malignant bone tumor in childhood and adolescents. It occurs frequently in the long bones and rarely in jaw and fingers. It accounts for around 5% of all pediatric malignancies. The standard treatment protocol of chemotherapy is a combination of doxorubicin, cisplatin, and methotrexate which have cardiotoxic effect [1]. Prior research work has thoroughly focused on left ventricular (LV) dysfunction in patients receiving cardiotoxic chemotherapy. However, little attention has been directed to show the effect of chemotherapy on right ventricular (RV) function. Diagnosis of RV dysfunction has been propelled to the forefront of research work as several studies substantiate the belief that, RV dysfunction is associated with increased morbidity and mortality in many cardiovascular diseases [2].

Assessment of RV function has been challenging. The right ventricle has a complex geometric shape which includes smooth muscular inflow, outflow, and a trabecular apical region; hence, no single s echocardiographic parameter is satisfactory for its evaluation [3]. Establishment of new echocardiographic techniques, e.g., two-dimensional speckle-tracking echocardiography (2DSTE) created the obstacles in RV imaging less difficult. 2DSTE is considered an important tool in assessing RV myocardial deformation, which is a powerful predictor of patient’s functional capacity and survival [4]. Many recent studied approved that RV global longitudinal strain (GLS) assessed by 2DSTE correlated well with RV function assessed by cardiac magnetic resonance imaging (CMRI) which is considered a well-known gold standard for RV assessment [5]. The aim of our study is to investigate the effect of anthracycline chemotherapy on right ventricular function by 2DSTE in children with osteosarcoma. Additionally, we aimed to highlight the role of 2DSTE in early detection of RV dysfunction in comparison to other usual echocardiographic modalities.

## Methods

This study was conducted on 100 patients with osteosarcoma in childhood (group 1) and another 100 ages matched healthy control persons (group 2). These patients were collected from multiple centers during the period from March 2017 to January 2019. All patients gave a written informed consent from their 1st degree relatives, and the study was approved by the local ethical committee. The study was in accordance with the principles of the Declaration of Helsinki II, and a code number was given for every patient pointed to his name, address, and telephone number.

### Inclusion and exclusion criteria

All included patients had been diagnosed to have osteosarcoma in the long bone, e.g., humerous and femur with normal LV function at baseline and on follow-up echocardiographic studies. All patients did not have a history of any other medical diseases, and patients with renal disorders, chronic pulmonary diseases, valvular heart disease, congenital heart disease, impaired LV function, or impaired LV GLS at any time of follow-up were excluded from the study.

### The protocol of chemotherapy

All patients received standard protocol of six cycles of chemotherapy (MAP), methotrexate (M) intravenous infusion 12 g/m^2^, doxorubicin (A) intravenous infusion 37.5 mg/m^2^ days 1, 2 cisplatin (P) intravenous infusion 60 mg/m^2^ days 1, 2 anthracycline cumulative dose: 450 mg/m^2^ as shown in Table 1 [6].

**Table 1:**
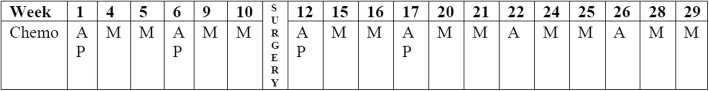
Standard protocol for chemotherapy in osteosarcoma

### Echocardiographic image acquisition and analysis

All patients had undergone echocardiographic examination at baseline, 10th week, 20th week, 29th week, and then follow-up 3 months after completion of the chemotherapy. Standard 2D echocardiographic examination was carried out using Vivid 7 or E 9; GE Vingmed Ultrasound, Horten, Norway, echocardiographic scanners, equipped with a 1.7-3.4 MHZ transducer (M3S probe). The frame rate was optimized < 90 and > 40 frames/s with ECG gating. At least 3 consecutive cine loops were stored for offline analysis using (Echo PAC version 112; GE Vingmed Ultrasound). The measurements and calculated formulas in the current study were performed according to the American Society of Echocardiography [7] and the European Association of Cardiovascular Imaging recommendation of chamber quantification [8]. During ECG recording, right ventricular fractional area change (RV FAC) was measured and calculated from RV-focused apical four-chamber view by tracing of RV endocardial borders both in systole and diastole without tracing of tricuspid valve leaflets or endocardial trabeculae with the usage of this formula.

RV FAC (%) = 100 × (RV end diastolic area (RV EDA) − RV end systolic area (RV ESA) ÷ RV EDA.

FAC < 35% is considered abnormal [7]. Tricuspid annular plane systolic excursion (TAPSE) was measured by placing an M-mode cursor through the tricuspid annulus and measuring the amount of longitudinal motion of the annulus at the peak systole in the standard apical four-chamber view. TAPSE < 17 mm is considered abnormal [9]. Right ventricular free wall longitudinal strain was measured offline by tracing endocardial walls of RV in RV-focused apical four-chamber view in around 10 points in one frame, and then, it will automatically have tracked all over cardiac cycles. Automatically, software divided RV into three parts basal, mid, and apical and then determined GLS of RV free wall. LV ejection fraction (LVEF) was estimated by measuring LVEDD and LVESD using M-mode. Two dimensional left ventricular longitudinal strain was measured by performing speckle tracking in the three apical views. Manual tracing of endocardial borders at the onset of systole was done, after which the software tracked the myocardial speckle pattern frame-by-frame. The width and shape of the region of interest (ROI) were adjusted manually. Subsequently, the software automatically divides the LV into 17 segments covering the entire myocardium [10]. Ten randomly selected studies were reassessed by the same operator shortly after the first assessment for assessment of interobserver variability. Ten randomly selected studies were reassessed by another operator for assessment of interobserver variability for all measured strain values. Patients with osteosarcoma (group 1) were further subdivided into two subgroups according to the result of RVGLS into two subgroups (subgroup 1A: patients with normal RVGLS) and (subgroup 1B: patients with decreased RVGLS) in different stages of chemotherapeutic course.

### Statistical analysis

Statistical analysis was done using SPSS 23, IBM, Armonk, NY, USA. Quantitative data were expressed as mean ± standard deviation. Qualitative data were expressed as absolute values and percentage. Student’s *t* test was used to test the significance between two groups in quantitative data, and chi-square (*X*^2^) test of significance was used to assess the difference between two qualitative data. Intraclass correlation coefficient (ICC) was used to assess interobserver and intraobserver variability. *P* value < 0.05 was considered statistically significant.

## Results

The present study included 200 participants, 100 patients with osteosarcoma in one of the long bones (group 1) and 100 ages matched healthy control persons with no history of any disease (group 2). The patients’ ages ranged from 9–13 years with a mean of 11.85 ± 2.09 years including 50 male patients and 50 female patients with no statistically significant difference than the control group, also there was no statistically significant difference regarding BSA, LV dimensions, left atrium and aortic dimensions, LVGLS, RVGLS, RV FAC, and TAPSE at baseline evaluation of the patients before receiving any chemotherapeutic drugs as shown in Table 2.

**Table 2:** Basal characteristics, echocardiographic results, and RV 2DSTE of the two groups

	Group 1 (*N* = 100) (patients with osteosarcoma)	Group 2 (*N* = 100) (control group)	*P* value
Age, years	11.85 ± 2.09	12.13 ± 1.87	0.320
Male gender, *n* (%)	50 (50.0%)	52 (52.0%)	0.777
BSA, m^2^	1.39 ± 0.07	1.40 ± 0.08	0.394
IVS, cm	0.49 ± 0.07	0.50 ± 0.08	0.571
PW, cm	0.50 ± 0.08	0.51 ± 0.09	0.320
LVEDD, cm	3.60 ± 0.30	3.65 ± 0.28	0.211
LVESD, cm	1.71 ± 0.32	1.69 ± 0.31	0.687
LVEF, %	67.7 ± 3.62	68.2 ± 3.52	0.353
LVGLS, %	− 23.77 ± 0.93	− 23.83 ± 0.91	0.670
LA diameter, cm	1.89 ± 0.21	1.85 ± 0.22	0.237
AO, cm	1.90 ± 0.18	1.87 ± 0.20	0.196
RVGLS, %	− 24.75 ± 0.53	− 24.64 ± 0.35	0.110
Apical RVS, %	− 24.91 ± 0.72	− 24.87 ± 0.66	0.684
Mid RVS, %	− 24.16 ± 0.64	− 24.11 ± 0.53	0.472
Basal RVS, %	− 24.65 ± 1.05	− 24.45 ± 0.92	0.172
RVS, cm/s	13.80 ± 0.66	13.79 ± 0.64	0.940
RVE, cm/s	13.61 ± 0.48	13.55 ± 0.49	0.455
RVA, cm/s	12.00 ± 0.57	11.92 ± 0.58	0.297
Tapse, cm	1.87 ± 0.15	1.88 ± 0.15	0.679
FAC, %	35.80 ± 1.03	35.79 ± 1.06	0.946

*BSA* body surface area; *IVS* interventricular septum; *PW* posterior wall; *LVEDD* left ventricular end diastolic dimension; *LVESD* left ventricular end systolic dimension; *LVEF* left ventricular ejection fraction; *LVGLS* left ventricular global longitudinal strain; *LA* left atrium; *AO* aorta; *RVGLS* right ventricular global longitudinal strain; *Apical, Mid, and Basal RVS* right ventricular strain; *RVS* right ventricular systolic wave; *RVE* right ventricular E wave; *RVA* right ventricular A wave; *Tapse* tricuspid annular plane systolic excursion; *FAC* fractional area change

On follow-up, on the 10th week, we found 7 patients had impaired RV strain (free wall, apical, basal, mid), so we divided the patients in group 1 into two subgroups 1A and 1B according to RV strain; group 1A included 93 patients with normal RVGLS and group 1B included 7 patients with decreased RVGLS. Ages of the patients and BSA were significantly higher in group 1B than group 1A; also RV strain values were significantly decreased in group 1B than the other group. However, another studied parameters, e.g., LV dimensions, LVGLS, RV TAPSE, and FAC showed no statistically significant difference between the two groups as shown in Table 3.

**Table 3:** Basal characteristics, echocardiographic results, and RV 2DSTE of group 1 subgroups at 10 weeks of receiving chemotherapy

	Group 1A (*N* = 93) (patients with normal RV strain)	Group 1B(*N* = 7) (patients with decreased RV strain)	*P* value
Age, years	11.66 ± 2.03	14.43 ± 0.53	0.001*
Male gender, *n* (%)	46 (49.5%)	4 (57.1%)	0.695
BSA, m^2^	1.38 ± 0.08	1.50 ± 0.01	0.001*
LVEDD, cm	3.56 ± 0.25	3.64 ± 0.24	0.427
LVESD, cm	1.79 ± 0.35	1.78 ± 0.26	0.973
LVEF, %	66.65 ± 4.03	66.71 ± 1.60	0.964
LVGLS, %	− 23.93 ± 0.81	− 23.65 ± 1.15	0.393
RVGLS, %	− 24.15 ± 0.96	− 18.43 ± 0.78	0.001*
Apical RVS, %	− 24.90 ± 0.75	− 17.42 ± 0.53	0.001*
Mid RVS, %	− 24.17 ± 0.67	− 17.57 ± 0.53	0.001*
Basal RVS, %	− 24.59 ± 1.04	− 19.00 ± 0.01	0.001*
Tapse, cm	1.85 ± 0.14	1.81 ± 0.11	0.448
FAC, %	35.86 ± 0.88	35.57 ± 0.54	0.395

*BSA* body surface area; *LVEDD* left ventricular end diastolic dimension; *LVESD* left ventricular end systolic dimension; *LVEF* left ventricular ejection fraction; *LVGLS* left ventricular global longitudinal strain; *RVGLS* right ventricular global longitudinal strain; *Apical, Mid and Basal RVS* right ventricular strain; *Tapse* tricuspid annular plane systolic excursion; *FAC* fractional area change

* = means significant

**Fig. 1:**
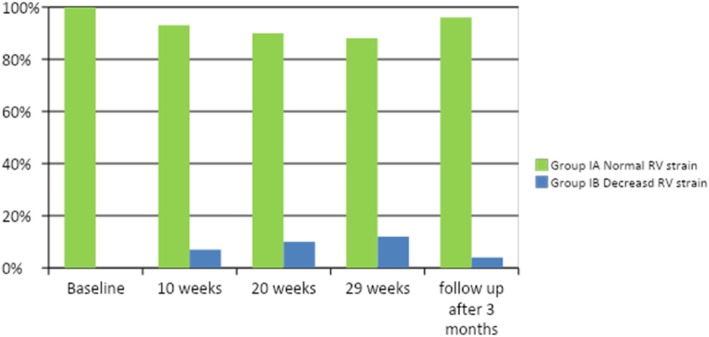
Percentage of patients with osteosarcoma with normal and decreased right ventricular global longitudinal strain in group 1 subgroups before, during and after recovery of chemotherapy

**Fig. 2:**
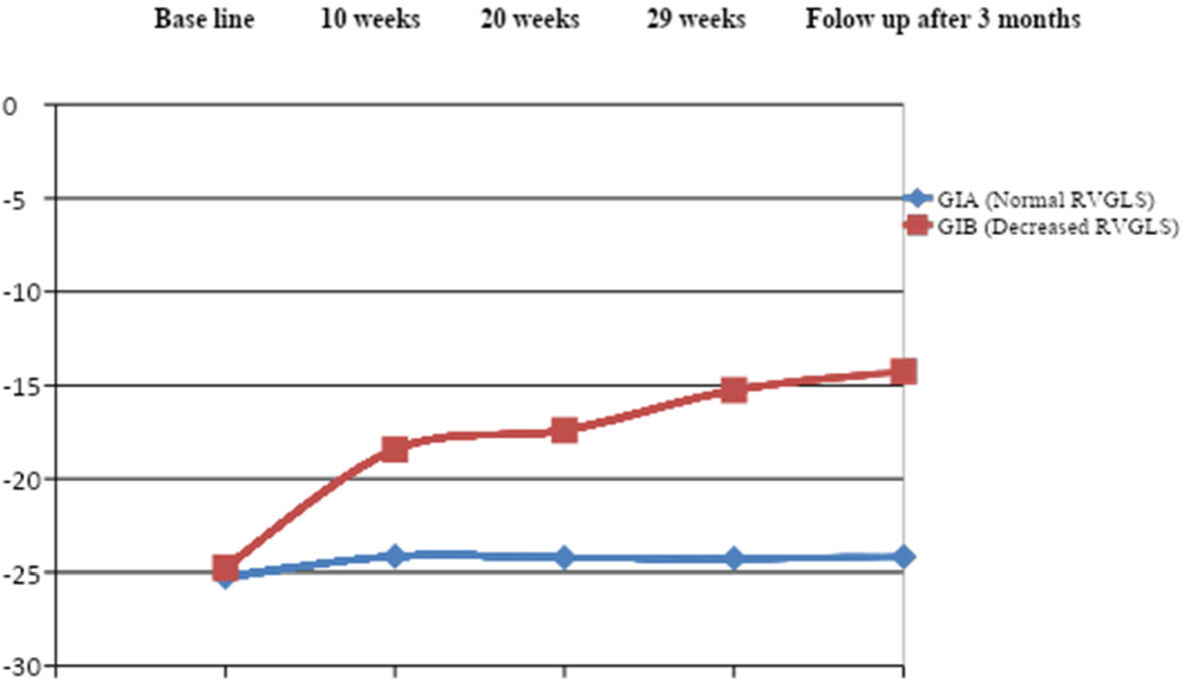
Right ventricular global longitudinal strain in group 1 subgroups before, during, and after recovery of chemotherapy

On the 20th week, the number of patents in group 1B was increased up to 10 patients with impaired RV strain, and we found that ages of the patients and BSA were significantly higher in the affected group (group 1B). On this time, TAPSE measured by conventional echocardiogram started to be affected and decreased significantly in the affected group. On the 29th week, patients with decreased RV strain in group 1B increased up to 12 patients with significantly higher ages and BSA in comparison to group 1A. On this time, TAPSE and RV FAC decreased significantly in group 1B than the other group as shown in Tables 4 and 5). Then, on follow-up 3 months after completion of the treatment protocol, 8 patients recovered from RV dysfunction and the number of patients in group 1B decreased to four patients only. These four patients had higher ages and BSA, with significant decrease in RV strain measurement (free wall, apical, mid, and basal), significantly depressed RV FAC, and significantly diminished TAPSE in comparison to group 1A as shown in Table 6, Figs. 1 and 2.

**Table 4:** Basal characteristics, echocardiographic results, and RV 2DSTE of group 1 subgroups at 20 weeks of receiving chemotherapy

	Group 1A (*N* = 90) (patients with normal RV strain)	Group 1B(*N* = 10) (patients with decreased RV strain)	*P* value
Age, years	11.56 ± 1.99	14.50 ± 0.52	0.001*
Male gender, *n* (%)	45 (50.0%)	5 (50.0%)	1.000
BSA, m^2^	1.38 ± 0.07	1.50 ± 0.01	0.001*
LVEDD, cm	3.62 ± 0.30	3.60 ± 0.21	0.789
LVESD, cm	1.97 ± 0.57	1.75 ± 0.26	0.225
LVEF, %	64.13 ± 7.51	66.50 ± 1.58	0.325
LVGLS, %	− 23.88 ± 0.86	− 23.60 ± 1.22	0.340
RVGLS, %	− 24.22 ± 0.89	− 17.40 ± 1.26	0.001*
Apical RVS, %	− 24.90 ± 0.76	− 16.40 ± 2.36	0.001*
Mid RVS, %	− 24.21 ± 0.64	− 17.01 ± 1.15	0.001*
Basal RVS, %	− 24.61 ± 1.05	− 18.20 ± 1.68	0.001*
Tapse, cm	1.85 ± 0.14	1.67 ± 0.24	0.044*
FAC, %	35.89 ± 0.88	35.10 ± 1.19	0.070

*BSA* body surface area; *LVEDD* left ventricular end diastolic dimension; *LVESD* left ventricular end systolic dimension; *LVEF* left ventricular ejection fraction; *LVGLS* left ventricular global longitudinal strain; *RVGLS* right ventricular global longitudinal strain; *Apical, Mid and Basal RVS* right ventricular strain; *Tapse* tricuspid annular plane systolic excursion; *FAC* fractional area change

* = means significant

**Table 5:** Basal characteristics, echocardiographic results, and RV 2DSTE of group 1 subgroups at 29 weeks of receiving chemotherapy

	Group 1A (*N* = 88) (patients with normal RV strain)	Group 1B (*N* = 12) (patients with decreased RV strain)	*P* value
Age, years	11.48 ± 1.94	14.58 ± 0.51	0.001*
Male gender, *n* (%)	45 (51.1%)	5 (41.7%)	0.538
BSA, m^2^	1.38 ± 0.07	1.48 ± 0.02	0.001*
LVEDD, cm	3.61 ± 0.30	3.62 ± 0.22	0.930
LVESD, cm	1.96 ± 0.57	1.87 ± 0.37	0.609
LVEF, %	64.00 ± 7.55	65.75 ± 1.35	0.427
LVGLS, %	− 23.85 ± 0.89	− 23.43 ± 1.13	0.142
RVGLS, %	− 24.27 ± 0.83	− 15.25 ± 1.86	0.001*
Apical RVS, %	− 24.89 ± 0.77	− 14.01 ± 3.13	0.001*
Mid RVS, %	− 24.24 ± 0.63	− 16.33 ± 2.50	0.001*
Basal RVS, %	− 24.62 ± 1.06	− 16.33 ± 2.46	0.001*
Tapse, cm	1.85 ± 0.14	1.47 ± 0.08	0.001*
FAC, %	35.89 ± 0.89	32.42 ± 2.27	0.001*

*BSA* body surface area; *LVEDD* left ventricular end diastolic dimension; *LVESD* left ventricular end systolic dimension; *LVEF* left ventricular ejection fraction; *LVGLS* left ventricular global longitudinal strain; *RVGLS* right ventricular global longitudinal strain; *Apical, Mid and Basal RVS* right ventricular strain; *Tapse* tricuspid annular plane systolic excursion; *FAC* fractional area change

* = means significant

**Table 6:** Basal characteristics, echocardiographic results, and RV 2DSTE of group 1 subgroups after 3 months of follow-up after receiving chemotherapy

	Group 1A (*N* = 96) (patients with normal RV strain)	Group 1B (*N* = 4) (patients with decreased RV strain)	*P* value
Age, years	11.73 ± 2.04	14.75 ± 0.50	0.004*
Male gender, *n* %	49 (51.0%)	1 (25.0%)	0.307
BSA, m^2^	1.39 ± 0.07	1.50 ± 0.01	0.006*
LVEDD, cm	3.63 ± 0.30	3.75 ± 0.28	0.449
LVESD, cm	1.96 ± 0.55	1.62 ± 0.25	0.229
LVEF, %	64.31 ± 7.31	65.75 ± 1.50	0.697
LVGLS, %	− 23.84 ± 0.88	− 23.40 ± 1.61	0.339
RVGLS, %	− 24.18 ± 0.91	− 14.25 ± 0.50	0.001*
Apical RVS, %	− 24.81 ± 1.02	− 12.01 ± 0.01	0.001*
Mid RVS, %	− 24.16 ± 0.67	− 14.75 ± 0.50	0.001*
Basal RVS, %	− 24.59 ± 1.03	− 14.50 ± 1.01	0.001*
Tapse, cm	1.83 ± 0.16	1.45 ± 0.05	0.001*
FAC, %	35.85 ± 0.87	31.00 ± 1.15	0.001*

*BSA* body surface area; *LVEDD* left ventricular end diastolic dimension; *LVESD* left ventricular end systolic dimension; *LVEF* left ventricular ejection fraction; *LVGLS* left ventricular global longitudinal strain; *RVGLS* right ventricular global longitudinal strain; *Apical, Mid and Basal RVS* right ventricular strain; *Tapse* tricuspid annular plane systolic excursion; *FAC* fractional area change

* = means significant

Expresses as intraclass correlation coefficients, RV strain and RV free wall strain had intraobserver variability of 0.94 to 0.98 (*P* < .001 for all) and interobserver variability 0.88 to0.98 (*P* < .001 for all).

## Discussion

Previous studies identified cardio toxic effects of anthracyclin on left ventricular function, to the best of our knowledge, no previous studies assessed RV dysfunction in children with osteosarcoma. We demonstrated that chemotherapeutic drugs adversely affect right ventricular strain in a short time after starting the protocol, despite other most echocardiographic RV echocardiographic indices of RV function remain within their normal range in the protocol period. This is in consistent with Tanindi et al. [11], who demonstrated impaired RV function during and after cardio toxic drugs in adults and children, but their assessment for RV function was done via tissue Doppler studies.

Regarding the incidence of RV dysfunction in cancer patient, in the current study, the incidence of RV dysfunction started by 7% and increased up to 12% at completion of the protocol. After follow-up after 3 months, 8% of patients were recovered, and only 4% had persistent impairment of RV global longitudinal strain. This incidence was in agreement to Mubraech et al. [12], who studied a long-term impact of cardio toxic treatment on RV function among adult’s lymphoma survivors who received stem cell transplantation, and they demonstrated that impairment of RV function was 6.2% but this study did not exclude patients with impaired LV function. Diversely, Ylanen et al. [13] found higher incidence of RV dysfunction around 27% when evaluating RV function by MRI in teenage survivors of childhood cancer. Essick et al. [14] demonstrated in their study that RV dysfunction occurs even in untreated cancer patients. This is could be explained by proinflammatory markers, reactive oxidative species, and neurohormonal changes in cancer patients. Additionally, Haarmark et al. [15] showed that RV systolic dysfunction occurs early in patients before starting chemotherapy. But unfortunately, those authors did not compare their results with healthy subjects. Consequently, we compared results of our selected patients with control group to exclude indirect cancer-related proinflammatory effects before starting chemotherapy. Furthermore, in the current study, we excluded patients with impaired LV GLS at any stage of treatment protocol, to exclude the indirect effect on RV remodeling that result from deterioration of LV mechanics.

Lencova et al. [16] demonstrated an experimental molecular study showing that there is a marked experimental asymmetry in the degree of molecular remodeling in response to chronic anthracyclin treatment, and they observed that LV myocyte being more affected than RV myocyte. Several studies demonstrated anatomical difference between the right and left ventricles, as right ventricle consists of two muscle layers instead of three layers in the left ventricle. They supposed that the right ventricle has a larger volume and so, less mass and it works against lower afterload [17]. Boczar et al. [18] studied RV in breast cancer patients who received anthracyclin, and they hypothesized that a thinner RV may be more sensitive to the toxicity of chemotherapy compared to the thicker LV but there are no other supportive data.

Regarding different parameters used in assessment of RV systolic function in the current work, we found that RV speckle tracking identified RV dysfunction earlier than other echocardiographic methods, followed by TAPSE, and the last affected parameter is RV FAC. This comes in agreement with Boczar et al. [18], who revealed that RV assessment by deformation technique is an important technique in identifying subclinical systolic dysfunction. Scanty studies investigate the influence of chemotherapy on RV. Belham et al. [19] observed no change in RV myocardial performance index in patients received low dose of anthracyclin. Also, Mubraech et al. [12] revealed impaired RV function in long-term lymphoma survivors, but they found that the most profound difference is in TAPSE. Also, Cottin et al. [20] reported no change in RV function after anthracyclin by multiple gated acquisitions. However, Yildirim et al. [21] found alterations in tissue Doppler velocities of RV at rest and dobutamine stress echocardiography. It is worthy to highlight that not all researchers agreed about the negative influence of chemotherapy on RV function. Belham et al. [18] studied 23 patients treated with low-dose anthracyclin and revealed no significant change in the RV Tei index after therapy. Moreover, Havsteen et al. [22] also did not find any reduction in RV ejection fraction after therapy with epirubicin in female patients with breast cancer.

## Conclusion

To the best of our knowledge, no prior researches have been evaluated direct toxic effect of anthracyclin chemotherapy in children with osteosarcoma on RV systolic function. We advocate the idea that chemotherapy could directly affect RV in the absence of any apparent negative effect on LV and although evident, the incidence of direct RV dysfunction may be less frequent than LV affection. RV 2DSTE was the best modality to detect early affection of RV function before a measurable impact on other RV traditional echo parameters.

### Limitations

1. Number of the patients in this study was relatively small, and we need large numbers to validate the results.
2. This work falls short of addressing the long-term effect of RV dysfunction on the prognosis of patients with osteosarcoma receiving anthracycline.

## Data Availability

All data and equipment were available at Tanta University Hospital and Al Azhar University Hospital.

## Abbreviations

2DSTE: Two-dimensional speckle-tracking echocardiography
CMRI: Cardiac magnetic resonance imaging
Doxorubicin (A): Cisplatin (P)
ECG: Electrocardiogram
FAC: Fractional area change
GLS: Global longitudinal strain
LV: Left ventricle
MAP: Protocol of methotrexate (M)
RV: Right ventricle
TAPSE: Tricuspid annular plane systolic excursion
